# A policy-maker’s perspective on ‘ECDC and EMCDDA guidance: prevention and control of infectious diseases among people who inject drugs’

**DOI:** 10.1186/1471-2334-14-S6-S11

**Published:** 2014-09-19

**Authors:** Joan Colom I Farran

**Affiliations:** 1 Programme on Substance Abuse, Public Health Agency of Catalonia, Autonomous Government of Catalonia, Barcelona, Spain

## 

In late 2011, the European Centre for Disease Prevention and Control (ECDC) and European Monitoring Centre for Drugs and Drug Addiction (EMCDDA) published *ECDC and EMCDDA Guidance: Prevention and Control of Infectious Diseases among People Who Inject Drugs *[1]. This document synthesised evidence-based good practices and expert opinion into a body of pragmatic recommendations for European policy-makers and programme planners.

*ECDC and EMCDDA Guidance* is a valuable resource for those who are addressing one of the most serious health problems related to illegal drug use: infectious diseases. The document focuses primarily on HIV, tuberculosis, hepatitis B and hepatitis C. It has the potential to encourage political decision-making based on scientific evidence rather than on ideological principles, and it may also lay the groundwork for more harmonised interventions within the European Union [2]. The purpose of this commentary is to highlight key elements of the *ECDC and EMCDDA Guidance* and to consider how guidance of this nature might be further strengthened in the future.

In my opinion, the two major contributions of the document are to identify the core values and principles that should guide interventions and to stress the importance of combining key interventions.

The core values and principles encompass issues such as client confidentiality, service accessibility, non-judgmental service provision, human rights and health promotion. It is important to recognise the centrality of these issues because people who use drugs are widely made the object of stigmatization and discrimination [3]. This is a relevant specific feature which distinguishes them from other types of patients. This fact can condition and limit the quality and coverage of care provided to drug users. If policies and drug services fully incorporate the core values and principles identified in the *ECDC and EMCDDA Guidance*, without any doubt this would greatly facilitate the implementation of the key interventions to prevent infectious diseases.

Regarding the importance of combining key interventions, efforts to prevent hepatitis C have made us keenly aware that the prevention measures suggested in the document will be most effective when they are integrated with each other and provided at good coverage levels [4]. Isolated interventions or integrated interventions that are not widely implemented will scarcely have any impact on public health, even though they may be used to justify questionable political decisions.

Additionally, the interventions described in *ECDC and EMCDDA Guidance* include three points that I wish to highlight.

First, the provision of sterile syringes and other drug consumption equipment should not be made conditional upon the exchange of used syringes. *ECDC and EMCDDA Guidance* states, “In order to achieve the goal of infectious disease prevention, easy access to needles and syringes should be promoted and the return of needles and syringes should be encouraged, but not absolutely required, in order to receive new supplies.” This guidance embodies the recognition that there is a need to go from exchange to distribution with the aim of achieving higher levels of coverage [5]. Parallel efforts should be made to recover the maximum number of syringes distributed, but this aim should never result in clients being restricted from acquiring the sterile syringes and other supplies that they need.

Second, too many opiate substitution services in Europe currently do not include syringe provision, or else people are required to quit using drugs in order to be allowed to access opiate substitution treatment. *ECDC and EMCDDA Guidance* observes that according to a meta-analysis of six United Kingdom-based studies [6], “the two interventions acted synergistically, reducing the odds of new [hepatitis C] infection by nearly 80% when a high coverage of needle and syringe programmes was provided for those who continued to inject while on opioid substitution treatment.” Simultaneously receiving substitution treatment and accessing clean equipment should be considered a key preventive strategy. Drug workers might seek to change the situation of people who still inject while receiving substitution therapy, but denying access to effective interventions is not the way to accomplish this goal.

Third, heroin-assisted therapy is briefly mentioned in the *ECDC and EMCDDA Guidance* as an effective opiate addiction treatment. In fact there are many studies proving its efficacy, especially for those failing with other substitution therapies [7]. I want to stress this from the *ECDC and EMCDDA Guidance* because there are still very few countries implementing it.

Regarding other points, I wish to present a somewhat different perspective. *ECDC and EMCDDA Guidance* highlights the strategy of integrating a range of services for people who inject drugs into a “one-stop shop” service delivery model. This approach has merit as a means of reaching as many drug users as possible and providing as many prevention activities as possible because patients referred to standard health services often get lost, especially when they are confronted with complex care pathways that are difficult navigate. However, I believe that if a drug user has the capacity to engage with health services that are utilised by other community members, he or she should be referred to those services. In that way, we normalize the situation and prevent social exclusion. Therefore, the “one-stop shop” approach should be reserved for clients who seem unlikely to otherwise access the services they need [8].

*ECDC and EMCDDA Guidance* mentions supervised injecting facilities as a service for reducing risk behaviour and overdoses. I consider these facilities to be a valuable resource for preventing the transmission of infectious diseases among socially excluded drug users, and in my opinion they should be emphasised much more in any updated future version of the guidelines. I am aware that research on those facilities is limited, but do you really need much research to prove that injecting inside these facilities is much safer than injecting elsewhere? (It is like needing to prove that jumping from a plane with a parachute is safer than jumping without one, is it not?)

Also for future editions of the guidelines, I recommend including strategies for preventing the transition from non-injecting routes of drug administration to injecting. Again, there is not much evidence on effective programmes, but this is because little research has been conducted on the topic. We do know, however, that injecting strongly increases the risks associated with drug consumption.

There is no doubt that *ECDC and EMCDDA Guidance* is a valuable tool for policy-makers aiming to curb infectious diseases among injecting drug users. In too many European countries, however, moral and ideological reasons are still being invoked to block the measures identified in the guidance, even though many of these measures are supported by strong scientific evidence of their effectiveness. That phenomenon surely does not occur in relation to any health condition other than drug addiction. Ultimately the success of the *ECDC and EMCDDA Guidance* and the impact on public health will depend on how far we the policy-makers are able to move away from moral and politically “correct” approaches.

## Competing interests

The authors declare that they have no competing interests.

## Declarations

This article has been published as part of *BMC Infectious Diseases* Volume 14 Supplement 6, 2014: Viral Hepatitis in Europe. The full contents of the supplement are available online at http://www.biomedcentral.com/bmcinfectdis/supplements/14/S6. The publication charges for this supplement were funded by AbbVie as an unrestricted grant to Rigshospitalet, the University of Copenhagen. AbbVie further funded the printing of the supplement with additional financial support from the Drug Prevention and Information Programme (DPIP) of the European Union.
